# Costs and health impact of delayed implementation of a national hepatitis B treatment program in China

**DOI:** 10.7189/jogh.12.04043

**Published:** 2022-07-08

**Authors:** Mehlika Toy, David Hutton, Jidong Jia, Samuel So

**Affiliations:** 1 Asian Liver Center, Department of Surgery, Stanford University School of Medicine, Palo Alto, California, USA; 2 Department of Health Management and Policy, University of Michigan, Ann Arbor, Michigan, USA; 3 Liver Research Center, Beijing Friendship Hospital, Capital Medical University, Beijing, China

## Abstract

**Background:**

Hepatitis B virus (HBV) infection is a leading public health problem in China. COVID-19 pandemic has interrupted the delivery of health care interventions worldwide, including HBV infection control.

**Methods:**

In this study, we used a Markov model to quantify the costs and population health impact of HBV treatment in China for the following scenarios: 1) current practice with only 17% of treatment eligible HBV infected adults receiving antiviral treatment; 2) reaching the World Health Organization (WHO) treatment target of 80% by 2030 with a steady increase in treatment rate beginning in 2022; and 3) the effect of a 1-5-year delay in meeting the 2030 WHO treatment target. A one-way as well as a probabilistic sensitivity analysis were conducted.

**Results:**

Without increasing antiviral treatment for treatment eligible HBV infected adults, the life-time health care costs for the estimated 89.2 million adults living with HBV in China is US$1305 billion and 10.8 million (12%) will die from HBV-related liver disease. Increasing treatment to achieve the WHO 80% target by 2030 would save US$472 billion and prevent 3.3 million HBV-related deaths. We estimated that a 1-year delay beyond 2030 in reaching the WHO 80% treatment target would likely lead to US$55 billion increase in future health care costs, and an additional 334 000 future deaths from HBV-related liver disease or cancer.

**Conclusions:**

Reaching the WHO 2030 with minimal delays would have an immense health and economic benefit. Implementing a national treatment program for HBV in China should be a key priority for policymakers.

In response to the United Nations’ 2030 Sustainable Development Goal to combat viral hepatitis, in 2016 the WHO issued the first global health sector strategy on viral hepatitis to eliminate viral hepatitis as a public health problem by 2030 [1]. Endorsed by all WHO member states, including China, the strategy set important prevention and vaccination targets to reduce hepatitis B virus transmission by 90% by 2030 and increase chronic hepatitis B (CHB) diagnosis and treatment from an estimated 9% and 8%, respectively, in 2015 to 90% and 80%, respectively, with the overall goal of reducing CHB-related liver disease and liver cancer deaths in the world.

Chronic hepatitis B infection is a major public health problem and the major cause of death from cirrhosis and liver cancer in China [1]. Although the current prevalence of CHB infection in children is very low due to China’s robust national newborn and infant hepatitis B immunization program, an estimated 86 million adults in China are hepatitis B surface antigen (HBsAg) positive that put them at risk for liver disease and liver cancer if they do not receive long-term monitoring and antiviral treatment when indicated [2]. In November 2017, China announced its national comprehensive action plan for viral hepatitis, which includes a plan to lower medicine costs and make more medical services and consultation on viral hepatitis available [3]. In November 2018, tenofovir and entecavir, the highly effective and low drug resistance antivirals were added to the China National essential drug list. Through collective pharmaceutical procurement, the annual drug pricing for generic tenofovir and entecavir has fallen to US$10 [4,5]. Currently, only an estimated 17% of treatment eligible HBV patients are receiving antiviral drug treatment [6].

The aim of this study was to quantify the health and economic impacts of reaching or exceeding the WHO 2030 hepatitis B treatment targets compared with the current treatment rates in China. The current COVID-19 pandemic has interrupted the routine delivery of health services worldwide [7]. Fear of COVID-19 transmission and the unknown novelty of the virus decreased inpatient and outpatient service utilization. We also modelled what the health and economic costs would be because of delays in reaching those targets due to the COVID-19 pandemic.

## METHODS

**Figure 1:**
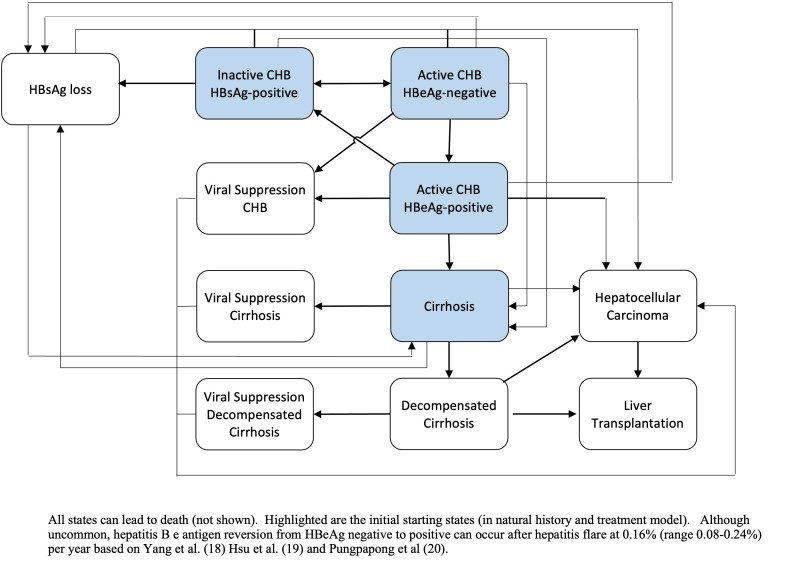
Markov schematic.

We used a Markov model to estimate the impact of hepatitis B treatment on health and economic outcomes [8,9]. The Markov (**Figure 1**) disease model simulates long-term outcomes, such as cirrhosis, hepatocellular carcinoma, and CHB-related death as patients with CHB infection move through various health states. Health states that are eligible for antiviral treatment are HBeAg-positive CHB patients with active hepatitis, HBeAg-negative CHB patients with active hepatitis, and patients with cirrhosis as defined by the 2018 AASLD guidelines for treatment of CHB [10]. Individuals who received treatment for active CHB and cirrhosis would have a lower risk of developing liver-related complications such as hepatocellular carcinoma and cirrhosis following disease progression rates derived from cohort studies and meta-analyses of HBV mono infected patients. Transitions in the Markov model are governed by age-specific (where available) disease progression estimates and treatment-related estimates and ranges that were collected from the literature (see key input **Table 1**). The Markov model was calculated using a 1-year time step, and implemented with TreeAge Pro 2021 (TreeAge Software, Williamstown, MA, USA).

**Table 1:** Key input variables

Variable	Base Case	Range	Distributions*	References
**Age/birth cohort**	≥20 y	20-80 y		
**HBsAg prevalence**	8.2%	5.0%-8.9%		See **Table 2**
**Percent adults receiving antiviral treatment**	17%	17%-19%	beta	Polaris [6]
**Percent of adults who are eligible for treatment**	40%	26%-40%		See **Table 2**
**Medical management and treatment costs:**				
**Antiviral drug costs per year**	US$ 10	10-36	gamma	WHO report 2019 [21]
**Annual monitoring†**	US$ 38.00	28-50	gamma	WHO report 2019 [21]
**Chronic hepatitis B**	US$ 3239	US$2592-3886	gamma	Zhang et al. 2016 [20]
**Cirrhosis**	US$ 5082	US$4066-6098	gamma	Zhang et al. 2016 [20]
**Decompensated cirrhosis**	US$ 6482	US$5186-7778	gamma	Zhang et al. 2016 [20]
**Hepatocellular carcinoma**	US$ 8569	US$6856-10 282	gamma	Zhang et al. 2016 [20]
**Liver transplantation 1^st^ year**	US$ 55 322	US$44 257-66 386	gamma	Zhang et al. 2016 [20]
**Liver transplantation 2^nd^ year**	US$ 33 907	US$27 126-40 689	gamma	Zhang et al. 2016 [20]
**Health state utilities:**				
**Active CHB**	0.77	0.76-0.87	beta	Zhang et al. 2021 [22]
**Cirrhosis**	0.75	0.74-0.89	beta	Zhang et al. 2021 [22]
**Inactive CHB**	0.99	0.95-1.0	beta	Zhang et al. 2021 [22]
**Decompensated cirrhosis**	0.68	0.67-0.78	beta	Zhang et al. 2021 [22]
**Hepatocellular carcinoma**	0.64	0.62-0.64	beta	Zhang et al. 2021 [22]
**Liver transplantation**	0.35	0.28-0.42	beta	Zhang et al. 2021 [22]
**HBsAg loss**	0.99	0.95-1.0	beta	Assumption
**Viral suppression**	0.99	(0.95-1.00)	beta	Assumption
**Transition (per year):**				
**From active CHB HBeAg-positive**				
**To HBsAg loss**	0.60%	(0.3-0.9)	beta	Ahn et al. 2005 [27]
**To cirrhosis**	1.60%	(1.3-1.9)	beta	Fattovich et al. 2008 [28]
**To HCC**	1.47%	(0.40-2.55)	beta	Thiele et al. 2014 [29]
**To HBV-related death**	0.11%	(0.09-0.14)	beta	Thiele et al. 2014 [29]
**To inactive**	7%	(4.0-10.0)	beta	Kanwal et al. 2005 [30]
**From active CHB HBeAg-negative:**				
**To HBsAg loss**	0.60%	(0.3-0.9)	beta	Ahn et al. 2005 [27]
**To active HBeAg-positive (reversion)**	0.16%	(0.08-0.24)	beta	Yang et al. 2012, Hsu 2002, Pungpagong 2007 [31-33]
**To cirrhosis**	2.80%	(1.3-4.3)	beta	Fattovich et al. 2008 [28]
**To HCC**	0.72%	(0.21-1.23)	beta	Thiele et al. 2014 [29]
**To HBV-related death**	0.11%	(0.09-0.14)	beta	Thiele et al. 2014 [29]
**To Inactive**	1.60%	(0.0-6.0)	beta	Kanwal et al. 2005 [30]
**From compensated cirrhosis**				
**To HBsAg loss**	0.60%	(0.3-0.9)	beta	Ahn et al. 2005 [27]
**To decompensated cirrhosis**	3.90%	(1.95-5.85)	beta	Lin et al. 2005 [34]
**To HCC**	3.16%	(2.58-3.74)	beta	Thiele et al. 2014 [29]
**To HBV-related death**	4.89%	(3.16-6.63)	beta	Thiele et al. 2014 [29]
**To viral suppression**	6.30%	(3.15-9.45)	beta	Chen et al. 2010 [35]
**From decompensated cirrhosis:**				
**To liver transplantation**	12.00%	(9.6-14.4	beta	Wang et al. 2013, Organ Transplantation Report China 2018 [36,37]
**To HCC**	7.10%	(3.55-10.65)	beta	Lin et al. 2005 [34]
**To HBV-related death**	15%	(7.50-22.5)	beta	Lin et al. 2005 [34]
**From HCC**				
**To liver transplantation**	4.70%	(3.7-5.6)	beta	Wang et al. 2013, Organ Transplantation Report China 2018 [36,37]
**To HBV-related death**	15.10%	(13.9-16.4)	beta	Thiele et al. 2014 [29]
**From viral suppression cirrhosis**				
**To HBsAg loss**	1%	(0.5-1.5)	beta	Ahn et al. 2005 [27]
**To HCC**	1.58%	(1.29-1.87)	beta	Thiele et al. 2014 [29]
**To HBV-related death**	2.44%	(1.58-3.31)	beta	Thiele et al. 2014 [29]
**From liver transplantation decompensated cirrhosis**				
**To HBV-related death year 1**	17%	(8.5-48.0)	beta	Burra et al. 2013 [38]
**To HBV-related death year 2+**	2.50%	(1.25-24.0)	beta	Burra et al. 2013 [38]
**From liver transplantation HCC**				
**To HBV-related death year 1**	16%	(8.0-48.0)	beta	Burra et al. 2013 [38]
**To HBV-related death year 2+**	2%	(2.0-25.0)	beta	Burra et al. 2013 [38]
**From inactive**				
**To HBsAg loss**				
**Age-group 40-49**	1.65%	(0.82-2.47)	beta	Chu et al. 2007, Chu et al. 2009 [39,40]
**Age-group 50+**	1.80%	(0.91-2.74)	beta	Chu et al. 2007, Chu et al. 2009 [39,40]
**To active CHB, HBeAg-negative**				
**Age-group 40-49**	2.80%	(1.4-4.1)	beta	Chu et al. 2007, Chu et al. 2009 [39,40]
**Age-group 50+**	2.00%	(1.0-3.0)	beta	Chu et al. 2007, Chu et al. 2009 [39,40]
**To cirrhosis**				
**Age-group 40-49**	0.07%	(0.034-0.102)	beta	Chu et al. 2007, Chu et al. 2009 [39,40]
**Age-group 50+**	0.15%	(0.052-0.202)	beta	Chu et al. 2007, Chu et al. 2009 [39,40]
**To HCC**	0.17%	(0.02-0.62)	beta	Rafetti et al. 2016 [41]
**From HBsAg loss**				
**To cirrhosis**	0.28%	(0.14-0.42)	beta	Chu et al. 2007, Chu et al. 2009 [39,40]
**To HCC**	0.09%	(0.045-0.136)	beta	Liu et al. 2014 [42]
**Transition estimates (per year) treatment**				
**From active CHB HBeAg-positive**				
**To HBsAg loss**	3%	(1.5-4.5)	beta	Terrault et al. 2018 [10]
**To Cirrhosis**	0	0		assumption
**To HCC**	0.44%	(0.12-0.765)	beta	(70% reduction) Papatheodoridis et al. 2015, Nguyen et al. 2019 [16,17]
**To HBV-related death**	0	0		assumption
**To drug resistance**	0.01%	(0.0-0.01)	beta	Heathcote et al. 2011, Lok et al. 2016, Tenney et al. 2009 [16,17]
**To viral suppression**	76%	(65.0-85.0)	beta	Terrault et al. 2018 [10]
**From active CHB HBeAg-negative:**				
**To HBsAg loss**	1%	(0.5-1.5)	beta	Terrault et al. 2018 [10]
**To cirrhosis**	0	0		Assumption
**To HCC**	0.22%	(0.063-0.369)	beta	(70% reduction) Papatheodoridis et al. 2015, Nguyen et al. 2019 [16,17]
**To HBV-related death**	0	0		assumption
**To drug resistance**	0.01%	(0.0-0.01)	beta	Heathcote et al. 2011, Lok et al. 2016, Tenney et al. 2009 [14,15,18]
**To viral suppression**	93%	(65.0-99.0)	beta	Terrault et al. 2018 [10]
**From compensated cirrhosis:**				
**To HBsAg loss**	1.70%	(0.85-2.55)	beta	Buti et al. 2015 [43]
**To decompensated cirrhosis**	1.80%	(0.90-2.70)	beta	(50% reduction)
**To HCC**	1.60%	(1.25-1.75)	beta	(50% reduction) Wong et al. 2013 [18]
**To HBV-related death**	2.40%	(1.58-3.30)	beta	(50% reduction)
**To viral suppression**	78%	(65.0-78.0)	beta	Wong et al. 2013 [19]
**To drug resistance**	0.01%	(0.0-0.01)	Beta	Heathcote et al. 2011, Lok et al. 2016, Tenney et al. 2009 [14,15,18]
**From decompensated cirrhosis**				
**To liver transplantation**	12.00%	(9.6-14.4)	beta	Wang et al. 2013, Organ Transplantation Report China 2018 [36,37]
**To HCC**	3.50%	(1.75-5.25)	beta	(50% reduction) Wong et al. 2013 [19]
**To HBV-related death**	7.50%	(3.75-11.25)	beta	(50% reduction)
**To viral suppression**	78%	(65.0-78.0)	beta	Wong et al. 2013 [19]
**To drug resistance**	0.01%	(0.0-0.01)	beta	Heathcote et al. 2011, Lok et al. 2016, Tenney et al. 2009 [14,15,18]
**From HCC**				
**To liver transplantation**	4.70%	(3.7-5.6)	beta	Wang et al. 2013, Organ Transplantation Report China 2018 [36,37]
**To HBV-related death**	15.10%	(13.9-16.4)	beta	Wong et al. 2013 [19]
**From viral suppression CHB**				
**To HBsAg loss**	1.50%	(0.07-2.2)	beta	Terrault et al. 2018 [10]
**To HCC**	0.06%	(0.03-0.09)	beta	(70% reduction) Papatheodoridis et al. 2015, Nguyen et al. 2019 [16,17]
**From viral suppression cirrhosis:**				
**To HBsAg loss**	1.50%	(0.07-2.2)	Beta	Terrault et al. 2018 [10]
**To HCC**	0.80%	(0.40-1.20)	Beta	(50% reduction)
**To HBV-related death**	1.20%	(0.60-1.80)	Beta	(50% reduction)
**From viral suppression decompensated cirrhosis:**				
**To HCC**	3%	(1.5-4.5)	beta	Jang et al. 2015 [44]
**To HBV-related Death**	6.10%	(3.05-9.15)	Beta	Jang et al. 2015 [44]
**from liver transplantation for decompensated cirrhosis**				
**To HBV-related death year 1**	17%-32%	(8.5-48.0)	Beta	Burra et al. 2013 [38]
**To HBV-related death year 2+**	2.50%	(1.25-24.0)	Beta	Burra et al. 2013 [38]
**Relative risk of death after liver transplant‡**	1.0	(0.5-1.5)	Normal	assumption
**From liver transplantation for HCC**				
**To HBV-related death year 1**	16%-39%	(8.0-48.0)	Beta	Burra et al. 2013 [38]
**To HBV-related death year 2+**	2%	(2.0-25.0)	Beta	Burra et al. 2013 [38]
**Gender**				
**Relative progression rates for females§**	0.5	(0.25-0.75)	Normal	Le et al. 2017, Guy et al. 2013, Cohen et al. 2016 [11-13]
**Fraction of chronic HBV cases that are male**	60%			Le et al. 2017 [11]

CHB – chronic hepatitis B, HBsAg – hepatitis B surface antigen, HBV – hepatitis B virus, HCC – hepatocellular carcinoma, y – year

*These are the distributions used for the probabilistic sensitivity analysis. The distributions are set such that the means are centered on the base-case value and the standard deviations of the distributions are set to match one quarter of the ranges specified in the “Range” column of this table. Parameters with no distribution identified were not varied in probabilistic sensitivity analysis.

†Annual monitoring is the total cost including bi-annual clinic visits and blood tests for ALT and annual HBV DNA level plus assuming 50% would receive additional HCC surveillance consisting of bi-annual liver ultrasound and AFP blood tests as recommended by AASLD [10]. Zhang et al. 2016 [20] (medical management costs inflated to 2022 costs).

‡Parameter used only for sensitivity analysis and applied to all liver transplantation states.

§A 50% reduction in disease progression estimates was applied for females Abbreviations: CHB – chronic hepatitis B, HBeAg – hepatitis B e antigen, HBsAg – hepatitis B surface antigen, HBV – hepatitis B virus, HCC – hepatocellular carcinoma

### Cohort and definitions

Our cohort of HBsAg positive adults is based on China population census by age and the age specific prevalence of HBsAg. The age-group specific distribution of adults with HBV in China by HBeAg and liver disease status is shown in **Table 2**. We estimated 89.2 million adults (ages 20+) are HBsAg-positive, and 36.2 million are eligible for treatment including 12.6 million with cirrhosis and 23.6 million with active hepatitis without cirrhosis. These estimates are consistent with the report by The Polaris study [5]. Those in the inactive CHB health state (hepatitis B carriers) are those who are HBsAg-positive with normal alanine aminotransferase (ALT) levels and no cirrhosis. Those with cirrhosis or active disease would be candidates for treatment. Following the recent AASLD guidelines [10], active hepatitis where treatment is indicated is defined by an elevation of ALT >2 upper limits of normal or evidence of significant fibrosis (≥F2) associated with ALT>upper limit of normal plus elevated HBV DNA above 2000 IU/mL for HBeAg-negative, and above 20000 IU/mL for HBeAg-positive individuals. Outcomes from the model included scenario specific lifetime treatment costs, quality-adjusted life-years (QALYs) and new cases of cirrhosis, decompensated cirrhosis, hepatocellular carcinoma, liver transplantations, and CHB related deaths. From these per-person results, we were able to calculate population-level outcomes. Simulations were undertaken separately for the 20-29, 30-39, 40-49, 50-59, 60-69, 70-79, 80+ age groups, and overall estimates were made by combining the age-specific results into weighted averages for lifetime costs and QALYs and then taking ratios of the average. We simulated CHB populations with a male to female ratio of 60:40 [11]. A 50% reduction in disease progression estimates was applied for females, based on recent sex-specific studies [11-13] Treatment effectiveness estimates were expressed as reductions in disease progression risk [14-19]. We assumed that effective viral suppression would reduce the risk for liver cancer risk in cirrhotic and non-cirrhotic patients by 50% and 70% respectively compared with natural history [16,17,19]. We assumed that patients would be treated with the lowest-cost drug, generic tenofovir or entecavir (US$ 10/y), but we explored the impact of drug cost ranging from US$ 10-36/y (**Table 1**). We assume among the 36.2 million HBV infected adults who are eligible for treatment, 17% or 6.15 million people living with hepatitis B in China are currently receiving hepatitis B antiviral treatment [6].

**Table 2:** Population level prevalence of chronic hepatitis B in China, by age and disease status

Age-group (years)	HBsAg prevalence*	# HBsAg-positive‡	#Active CHB (without cirrhosis)†	#Cirrhosis†	Total needing treatment†
20-29	5.0%	9 247 151	3 206 290	647 301	3 853 591
30-39	8.6%	19 061 836	5 198 802	1 906 184	7 104 986
40-49	8.5%	18 960 188	4 416 520	1 896 019	6 312 539
50-59	8.9%	19 224 191	4 147 538	3 652 596	7 800 134
60-69	8.9%	13 318 720	3 891 414	2 397 370	6 288 784
70-79	8.9%	6 659 360	1 945 707	1 531 653	3 477 360
80+	8.9%	2 764 263	807 652	635 780	1 443 432
Total	8.2%	89 235 710	23 613 923	12 666 902	36 280 826

CHB – chronic hepatitis B, HBsAg – hepatitis B surface antigen

*Liang X et al. 2009 [45], Cui FQ et al. 2017 [46]

†Toy et al. 2014 [47]

‡According to 2020 census.

### Scenarios

We assessed the population health impact and cost-effectiveness for China in reaching or exceeding the WHO 2030 hepatitis B treatment target of 80% among adults eligible for treatment. And the costs of delay in meeting the 2030 treatment target by 1-5 years.

We evaluated several scenarios of how achieving the WHO targets might affect the economic and health outcomes related to hepatitis B. In the **current practice scenario**; we assume that 6.15 million or 17% of the total treatment eligible cohort is being treated and the remainder of the cohort follows the natural history of disease. In the **Meet WHO 2030 scenario**; we assume that the 80% treated target will be reached in 8 years (with a gradual increase of 7.9% per year between 2022 to 2030). In the **Exceed WHO 2030 scenario**; we assume that a 90% treated target will be reached by 2030 (with a gradual increase of 9.1% per year between 2022 to 2030). We modelled the health outcome including cases of cirrhosis, decompensated cirrhosis, hepatocellular carcinoma, liver transplants, and hepatitis B related deaths. We combined this into overall cost and quality-adjusted life-years (QALYs).

We also evaluate scenarios of delays in meeting the WHO 2030 goals. We evaluate the health and economic impacts of a 1-year, 2-year, 3-year, or 5-year delay.

### Cost and utility estimates

We used medical management costs for CHB and other related costs from a study by Zhang et al. [20] which was a nationwide survey of HBV associated economic burden in China. Since our analysis was from a third-party payer perspective, we chose to only take direct medical fees from the Zhang et al. study. The drug and monitoring costs were obtained from the WHO implementation progress of the regional action plan for viral hepatitis in the Western Pacific 2016-2018 report [21]. All costs were inflated to 2022 prices using China National Healthcare Index from National Bureau of Statistics of China and converted to US dollars. The utility estimates were obtained from a recent multicenter study [22] that measured the health-related quality of life and health utility value of patients with hepatitis B related disease in China. Costs and QALYs were discounted at a rate of 3% per year to turn future costs and QALYs into a present value equivalent.

### Sensitivity analyses

Annual disease progression probabilities and ranges (high and low values) were collected from the literature. We used one-way sensitivity analysis to determine the parameters that had the greatest impact on the results. We also conducted a probabilistic sensitivity analysis varying all parameter values simultaneously (by drawing them from distributions defined in **Table 1**) to evaluate the impact of overall parameter uncertainty on outcomes.

## RESULTS

**Figure 2:**
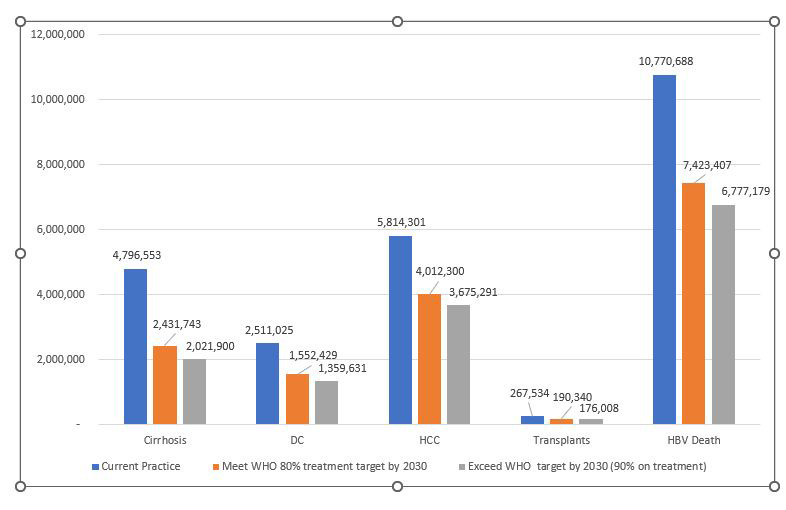
Comparing the cumulative deaths and liver complications of current practice with meeting the WHO treatment target of 80% and at 90% by 2030.

Under the current practice scenario where only 17% of treatment eligible infected patients are receiving antiviral drug treatment for hepatitis B, we projected among the 89.2 million HBsAg positive adults in China, over their lifetime, 4.80 million will develop cirrhosis, 2.51 million will develop decompensated cirrhosis, 5.81 million will develop hepatocellular carcinoma (HCC), 268 thousand will receive liver transplant treatment and 10.77 million (12%) will die from HBV-related liver disease or liver cancer (**Figure 2**). The estimated life-time health care costs for current practice is US$1305 billion and will result in 457.9 million QALYs (**Table 3**).

**Table 3:** Life-time health and economic impact (95% confidence interval) of a national hepatitis B treatment program in China

Scenario	Costs (billions in US$)	QALYs (millions)	New cases of cirrhosis	Decompensated cirrhosis	Hepatocellular carcinoma	Transplant	HBV death
Current practice (17% on treatment)	1305 (1123-1483)	458 (433-479)	4 796 553 (3 508 293-6 326 104)	2 511 025 (1 277 793-3 714 334)	5 814 301 (4 529 633-7 319 839)	267 534 (135 119- 472 388)	10 770 688 (8 988 219-13 070 264)
	**Savings**	**Gains**	**Averted**	**Averted**	**Averted**	**Averted**	**Averted**
Meet WHO 80% treatment target by 2030	472 (392-558)	52 (46-61)	2 364 810 (1 708 390-3 143 964)	958 596 (423 298-1 516 309)	1 802 001 (1 249 209- 2 389 372)	77 194 (23 849-141 677)	3 347 281 (2 729 967-4 052 340)
Exceed WHO target by 2030 (90% on treatment)	559 (467-661)	62 (55-73)	2 774 653 (2 006 479-3 687 065)	1 151 394 (507 223-1 825 168)	2 139 010 (1 484 638-2 844 144)	91 526 (27 665-168 903)	3 993 509 (3 254 458- 4 865 941)

CI – confidence interval, QALYs – quality-adjusted life years, US$ –US dollar

Compared with current practice, if treatment is increased at an incremental rate of 7.9% per year between 2022 and 2030 to meet the WHO Target of 80% on treatment by 2030, it will reduce new cases of cirrhosis by 2.36 million (49.30%), new cases of decompensated cirrhosis by 959 thousand (38.2%), new cases of HCC by 1.80 million (31.0%), new liver transplant cases by 77 thousand (29.9%), and HBV-related deaths by 3.35 million (31.1%) (**Table 3**). Compared with current practice, meeting the 2030 WHO HBV treatment target will result in US$472 billion in health care savings and an additional 52 million QALYs gained, compared with current practice. If the treatment target were exceeded and 90% were treated by 2030, compared with current practice the savings would rise to US$559 billion with an additional 62 million QALYs gained, and 4.0 million deaths averted.

**Figure 3:**
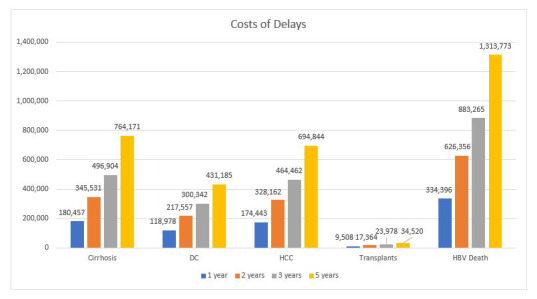
Health outcomes due to the delay in achieving WHO goals DC – decompensated cirrhosis, HCC – hepatocellular carcinoma.

We estimated that a 1-year delay in achieving the 80% treatment goal by 2030 would cost US$55 billion and lead to 7 million QALYs lost with 334 thousand additional HBV-related deaths (lifetime) (**Table 4** and **Figure 3**).

**Table 4:** Life-time costs and health impact (95% confidence interval) of delayed implementation of a national hepatitis B Treatment program in China to meet the WHO 80% treatment target by 2030

Delay (years)	Costs, billions in US$	QALYs lost, millions	Cirrhosis	DC	HCC	Transplants	HBV death
1	55 (46, 63)	7 (6, 8)	180 457 (126 719, 256 890)	118 978 (51 918, 190 856)	174 443 (121 289, 244 498)	9508 (5039, 16 203)	334 396 (265 413, 433 860)
2	102 (85, 118)	13 (11, 15)	345 531 (242 680, 490 346)	217 557 (95 098, 346 268)	328 162 (226 519, 458 623)	17 364 (8562, 29 366)	626 356 (498 787, 807 105)
3	144 (120, 165)	18 (15, 21)	496 904 (349 138, 703 945)	300 342 (131 511, 474 611)	464 462 (318 806, 647 093)	23 978 (10,853, 40 897)	883 265 (705 561, 1 131 153)
5	211 (176, 244)	26 (22, 30)	764 171 (537 699, 1 078 651)	431 185 (189 354, 675 385)	694 844 (473 330, 967 893)	34 520 (13 982, 60 054)	1 313 773 (1 055 395, 1 671 347)

CI – confidence interval, DC – decompensated cirrhosis, HCC – hepatocellular carcinoma, QALYs – quality adjusted life years, US$ – US dollar

### Sensitivity Analysis

The results of the one-way sensitivity analysis on the costs savings, QALYs gained, and HBV-related deaths averted by achieving the WHO goals are found in Figures S1a, S1day, and S1g in the **Online Supplementary Document**. These results show that the discount rate was influential when evaluating the overall impacts, however, there were no cases where achieving the WHO targets would be bad for health or add costs. Similarly, the results of the one-way sensitivity analysis on the additional costs associated with delay are shown in Figures S1b-c, S1e-f, and S1h-I in the **Online Supplementary Document**. There were no cases where a delay would be beneficial in terms of saving costs, adding QALYs, or averting deaths. The Monte Carlo simulation results showed similar findings (Figures S2a-2b in the **Online Supplementary Document**).

## DISCUSSION

We estimated that among the 89.2 million people living with hepatitis B in China, about 36.3 million or 40.7% are eligible for treatment including 12.7 million with cirrhosis and 23.6 million with active hepatitis. Assuming a gradual annual 7.9% increase in treatment rate from 2022 to 2030, our model projects it would prevent 1.8 million cases of HCC and 3.3 million HBV-related deaths at a saving of US$472 billion in future health care costs. Current WHO data shows that hepatitis B and C services, as well as HIV testing and prevention, are among the most frequently disrupted services caused by the COVID-19 pandemic [23,24]. In this study, we modelled the health and economic impact of a delay in increasing hepatitis B treatment to reach the WHO treatment target of 80% by 2030 in China. Our study suggests that a one-year delay beyond 2030 in reaching the 80% treatment target would lead to 334 thousand deaths from HBV-related liver disease and US$55 billion in future health care costs.

Apart from the disruptions that are likely caused by the COVID-19 pandemic, several factors that contributed to the delay in scaling up the national hepatitis B treatment program in China have been discussed previously [25,26]. The initial high cost of brand entecavir and tenofovir and training of health care workers in the management and treatment of hepatitis B is one barrier. Another barrier is unlike HIV, the governance system of viral hepatitis control is fragmented in China. There is no specific single department or unit within China National Health Commission (NHC) or China Centers for Disease Control and Prevention (CDC) to plan and lead the national effort to increase hepatitis B diagnosis and treatment [25]. Although recommended HBV drugs have been included in the National List of Reimbursable Medicines (NLRM) since February 2017, the actual reimbursement rates vary greatly across China due to the decentralized management and risk pooling across Chinese health insurance schemes [25]. According to a study [25] that summarized China achievements related to viral hepatitis, the policy poses challenges in achieving universal hepatitis treatment.

Although we used the best possible data that we could find for our analysis, our modelling study had several limitations. We only assessed the costs of hepatitis B management including the health care costs for longterm monitoring of HBsAg positive individuals and treatment including antiviral drug treatment and treatment of disease complications including liver cancer and liver transplantation. We did not include the costs of HBsAg screening to increase the diagnosis of those living with hepatitis B who are not aware of their infection. This study is taken from a thirdparty payer prespective and not the societal perspective. We did not include programmatic costs such as outreach and education to increase the treatment rate incrementally per year. We assume to reach the WHO 2030 target 80% of those eligible would be receiving treatment. Although there is no cure for chronic hepatitis B, the low risk for drug resistance antiviral medications, entecavir and tenofovir, are highly effective in treating liver inflammation to prevent disease progression and can even reverse fibrosis and cirrhosis to reduce the risk of liver cancer. We assume that the patients will be taking generic entecavir or tenofovir.

## CONCLUSIONS

Reaching the WHO 2030 treatment target of 80% would have a huge health and economic benefit. It would mean approximately 30 million treatment eligible HBV infected patients with or without cirrhosis would receive antiviral therapy by 2030. COVID-19 pandemic has likely delayed national HBV treatment efforts. Ensuring to implement a national treatment program without a delay in China should be a key priority for policymakers.

## Additional material


Online Supplementary Document
