# Supplementary material for: Costs and health impact of delayed implementation of a national hepatitis B treatment program in China
Source: J Glob Health. 2022 Jul 8;12:04043. doi: 10.7189/jogh.12.04043 (PMC9260492; doi:10.7189/jogh.12.04043)

Supplementary

Figure S1. Tornado Diagrams

Figure S1a: Cost-Savings for Goal of 80% by 2030

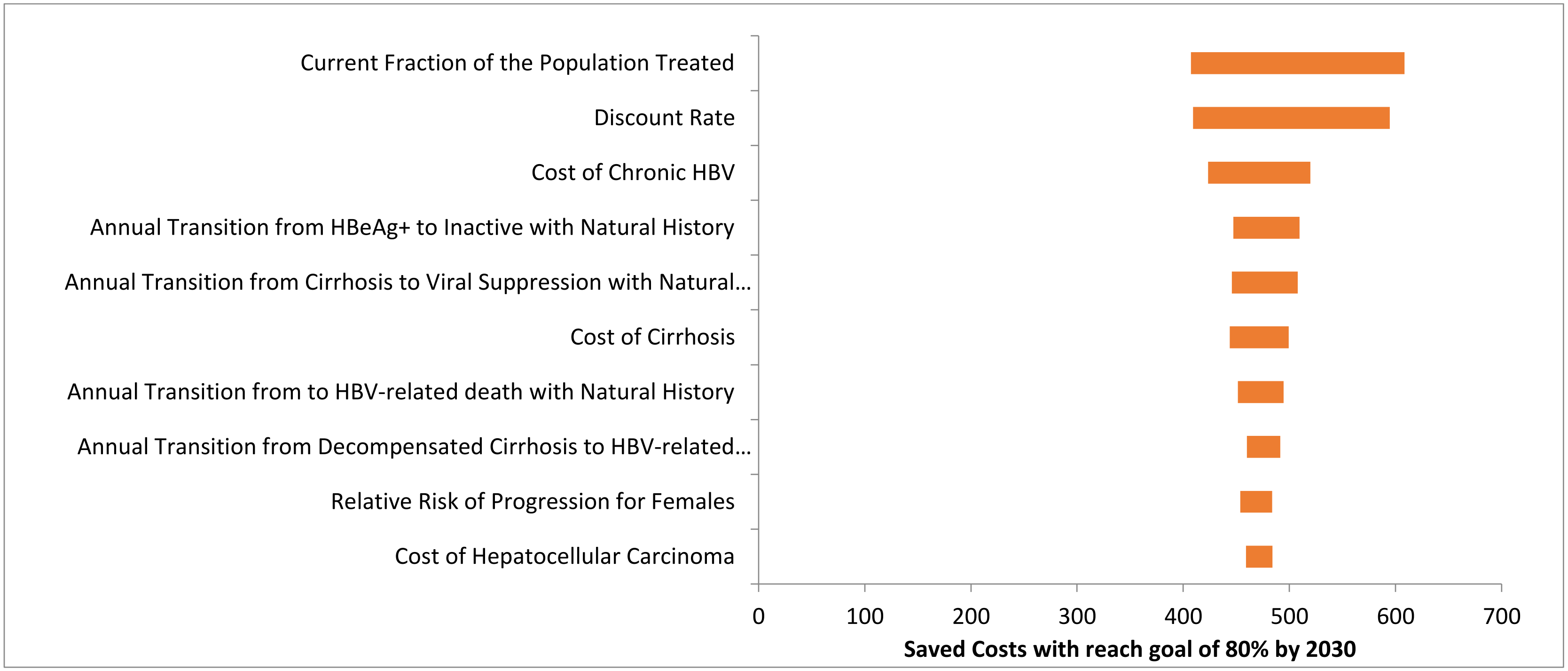

Figure S1b: Additional Costs 1-year delay

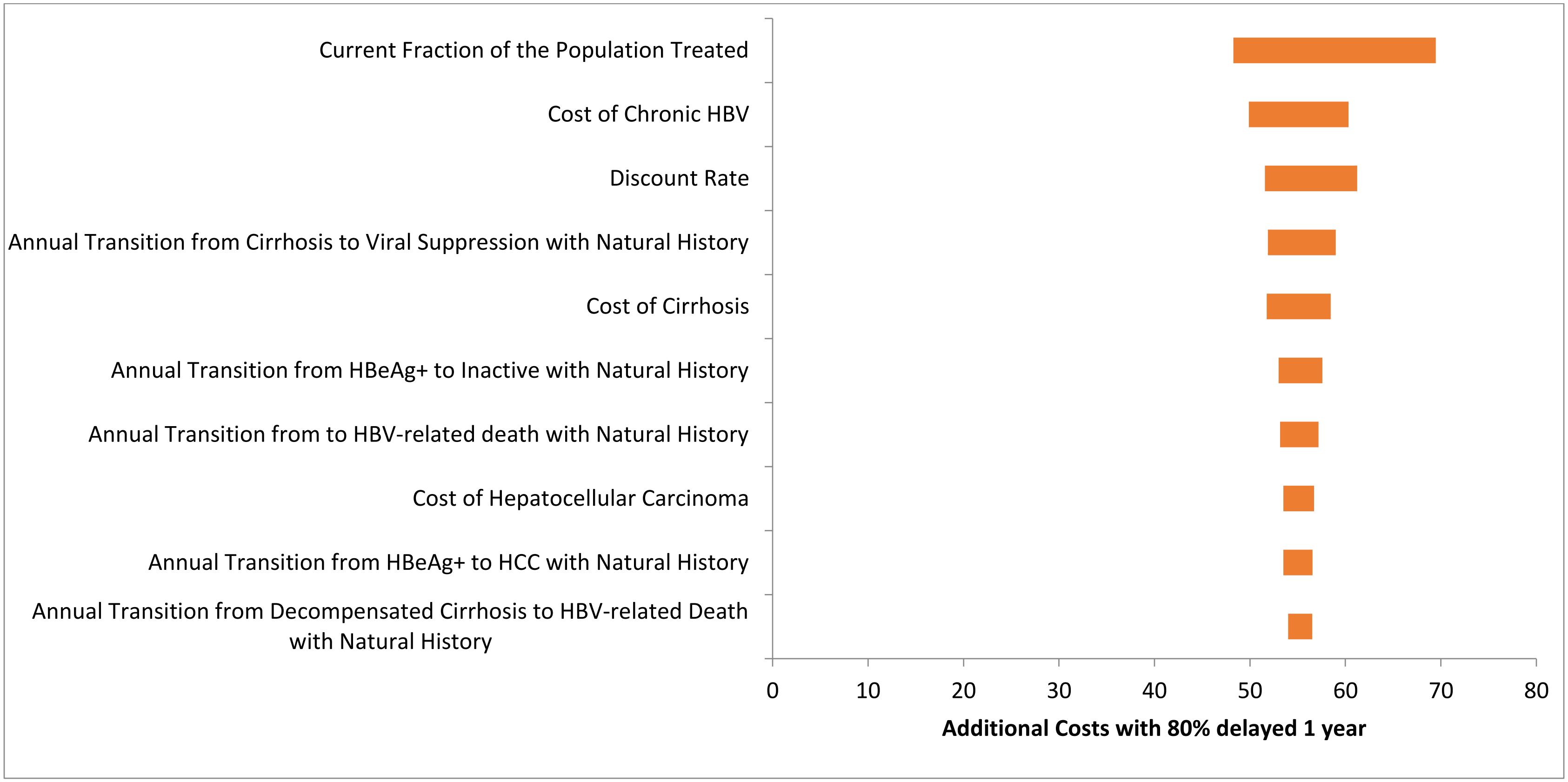

Figure S1c: Additional Costs for 5-year delay

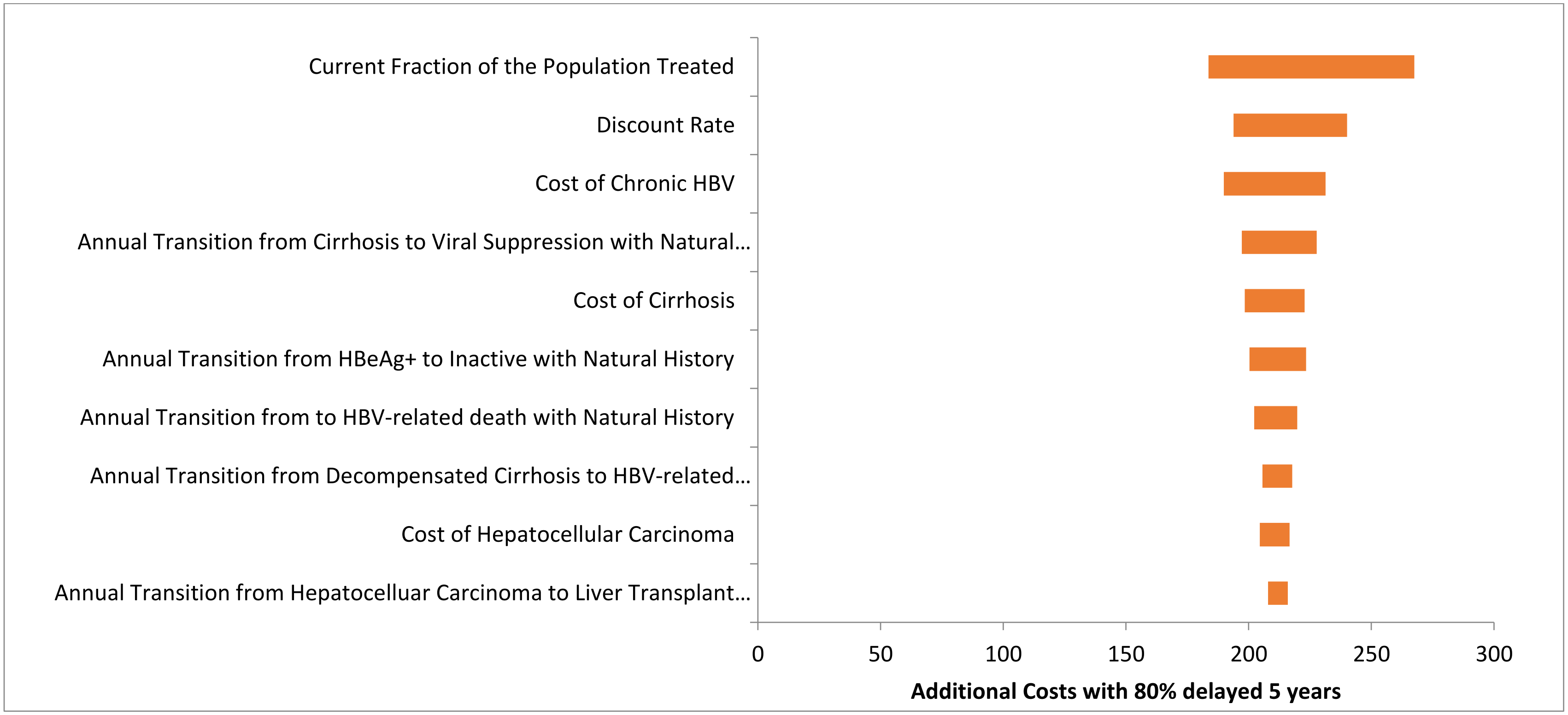

Figure S1d: QALYs gained by achieving WHO goals in 2030

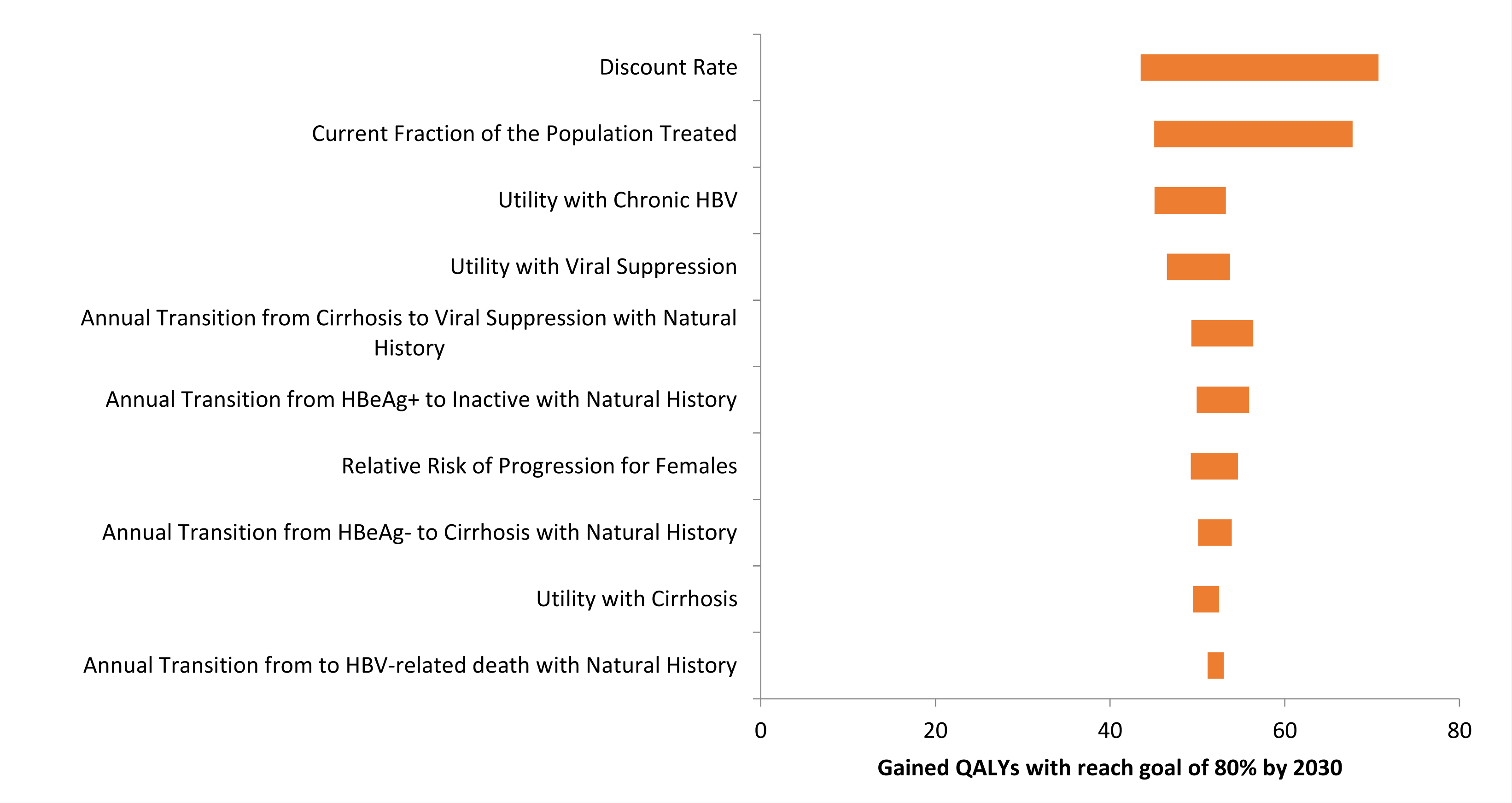

Figure S1e: QALYs lost from 1-year delay

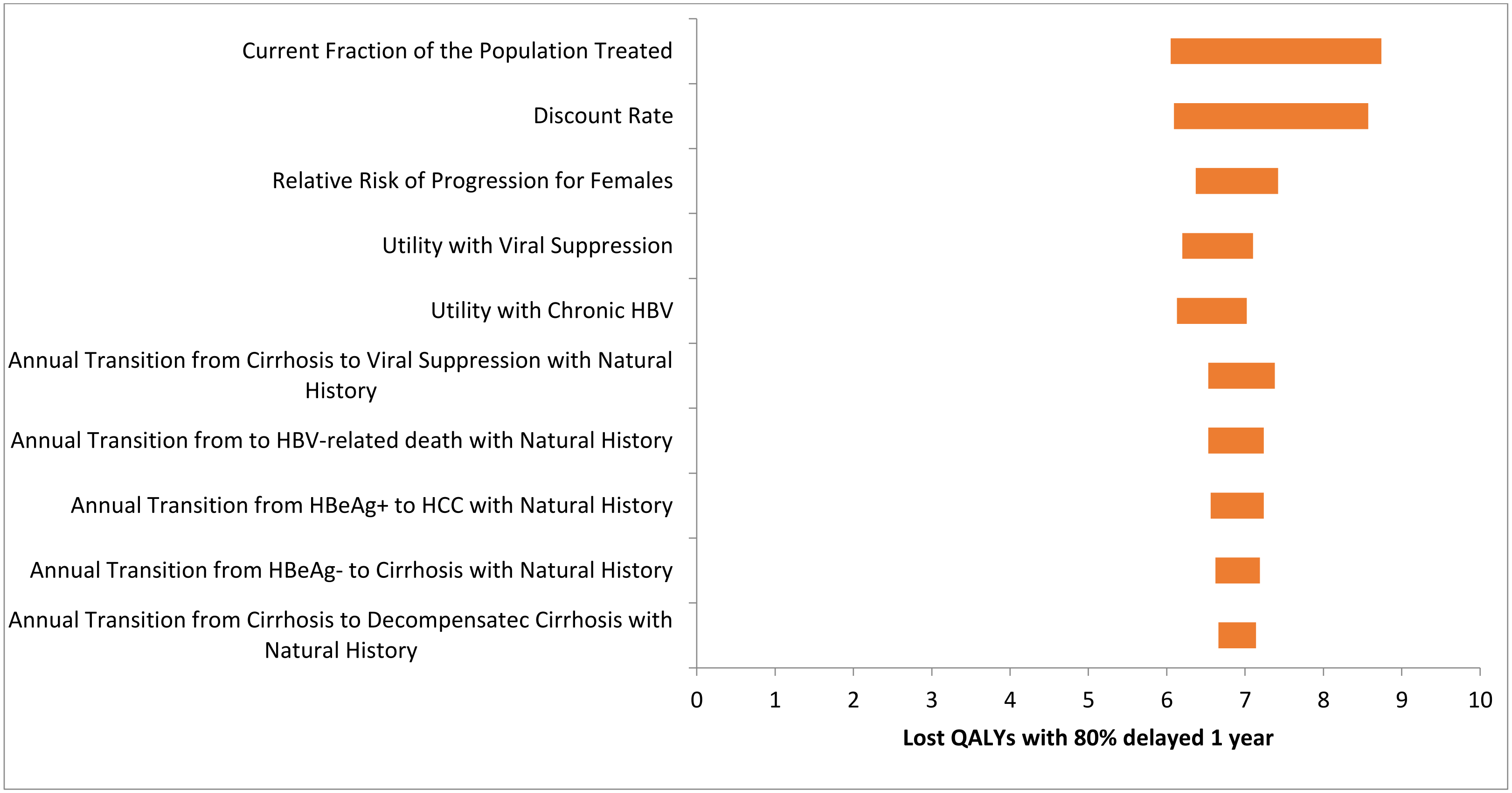

Figure S1f: QALYS lost from 5- year delay

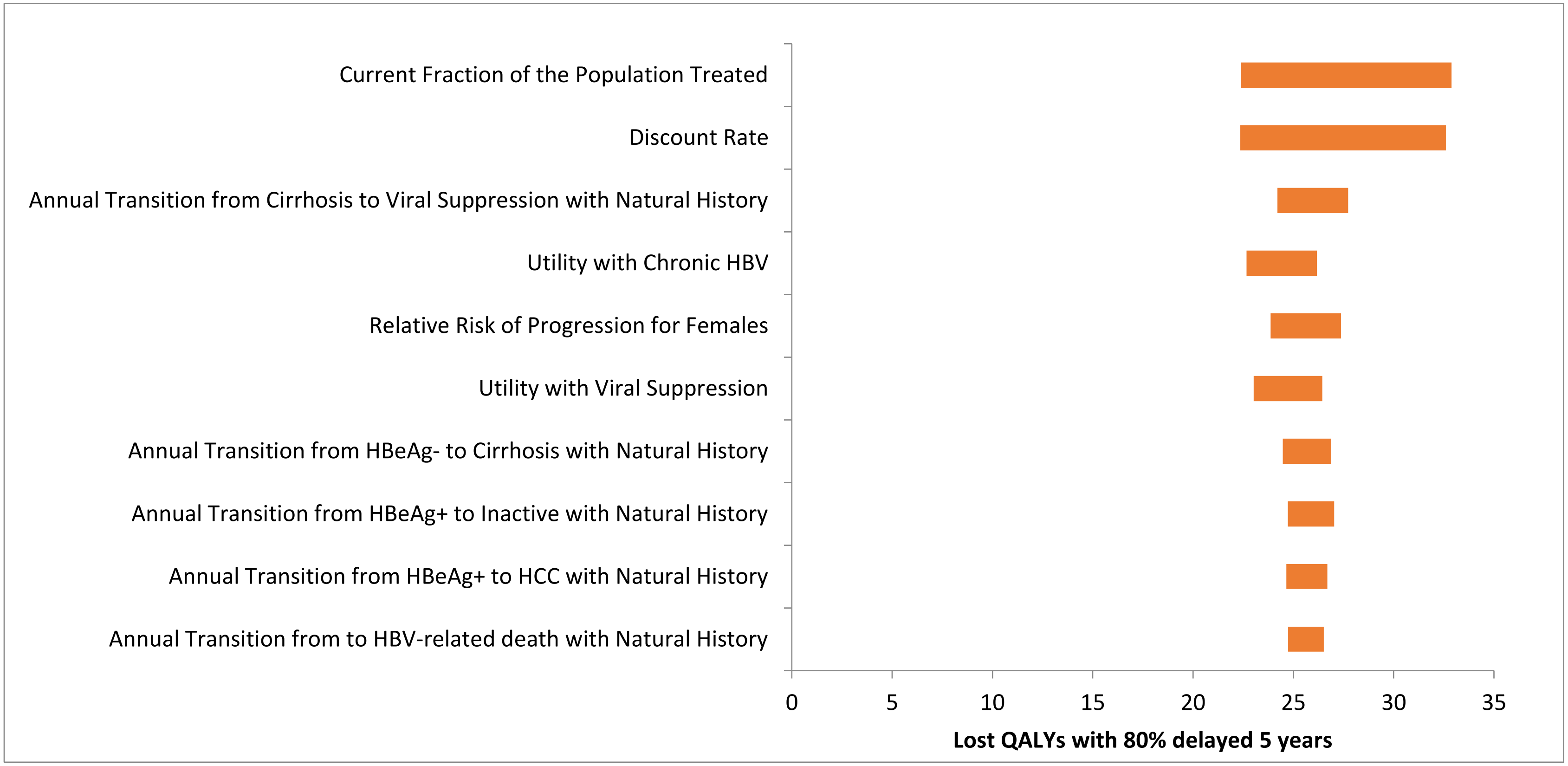

Figure S1g: Averted HBV-related Deaths achieving WHO goals

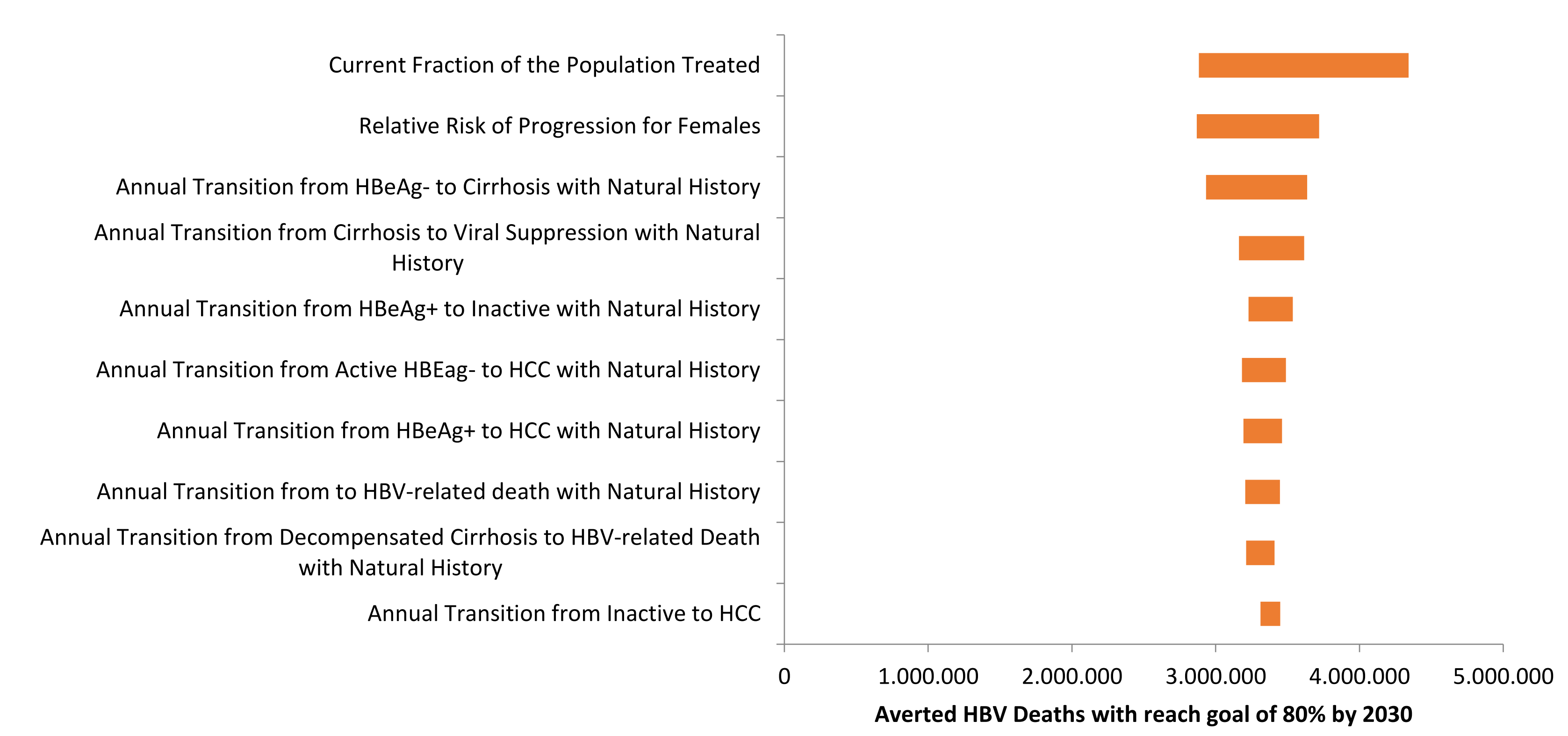

Figure S1h: Additional HBV-related Deaths with 1-year Delay

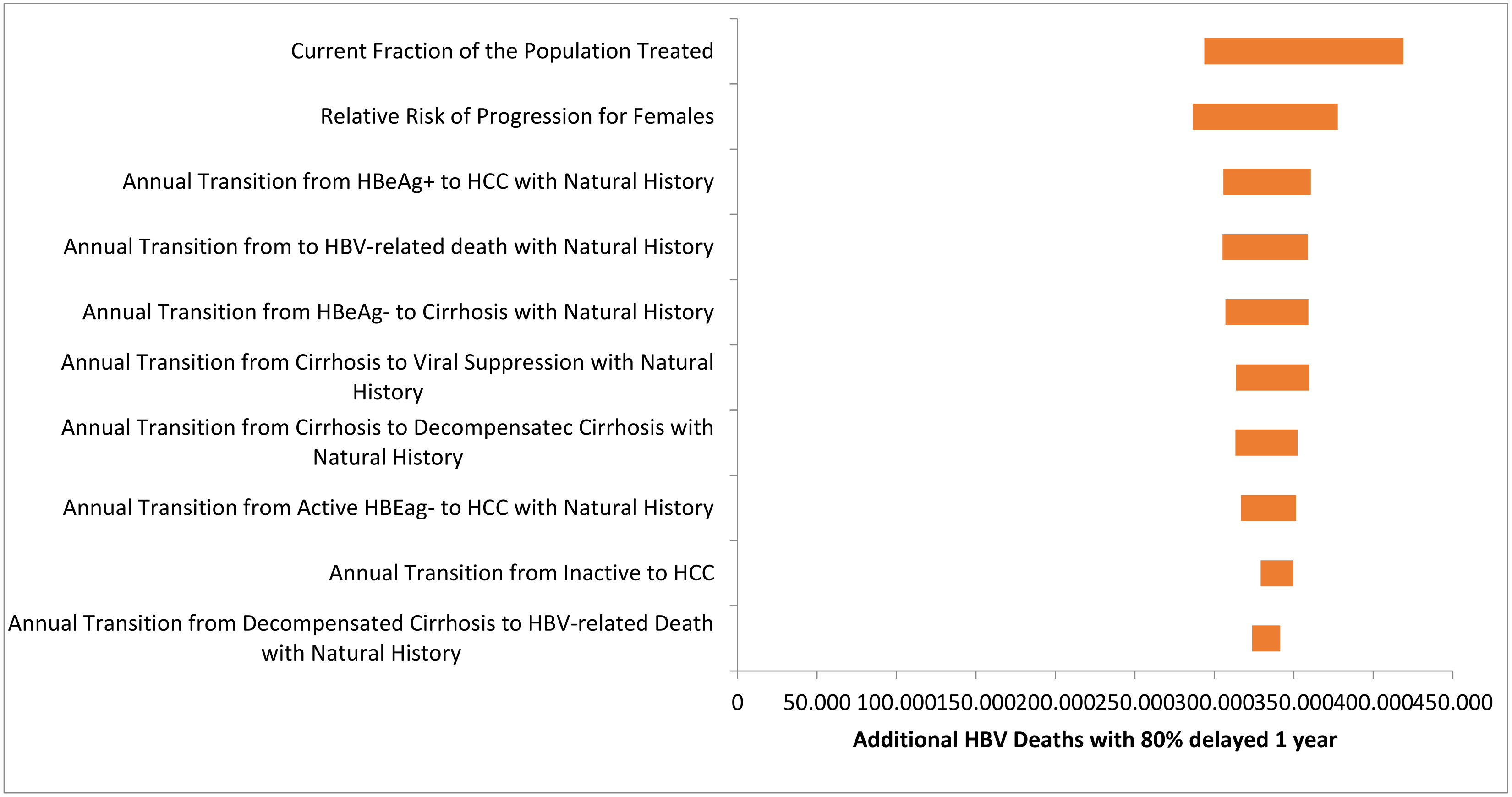

Figure S1i: Additional HBV-related Deaths with 5-year Delay

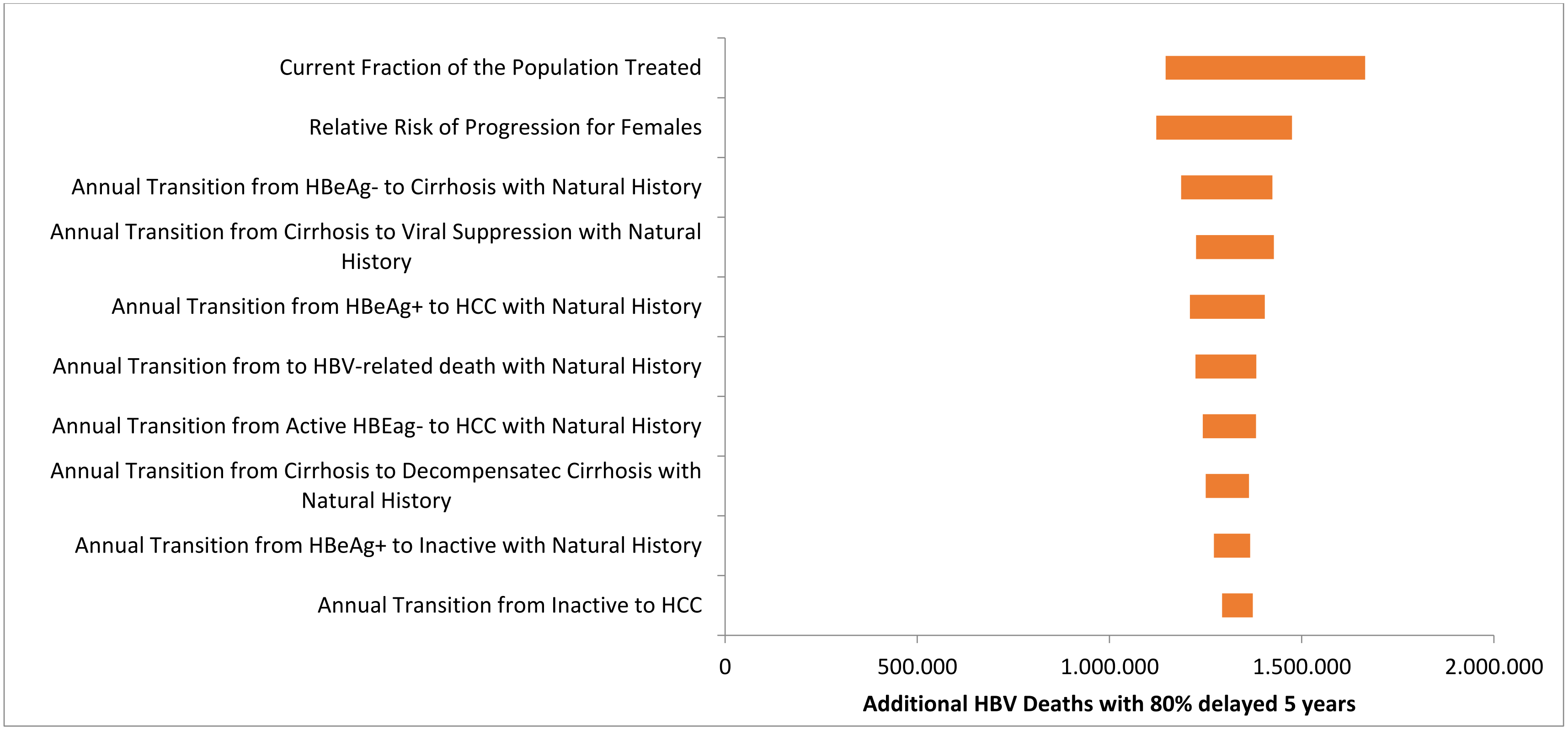

Appendix Figure S2: Results of the Probabilistic Sensitivity Analysis

Appendix Figure S2a: Cost Savings

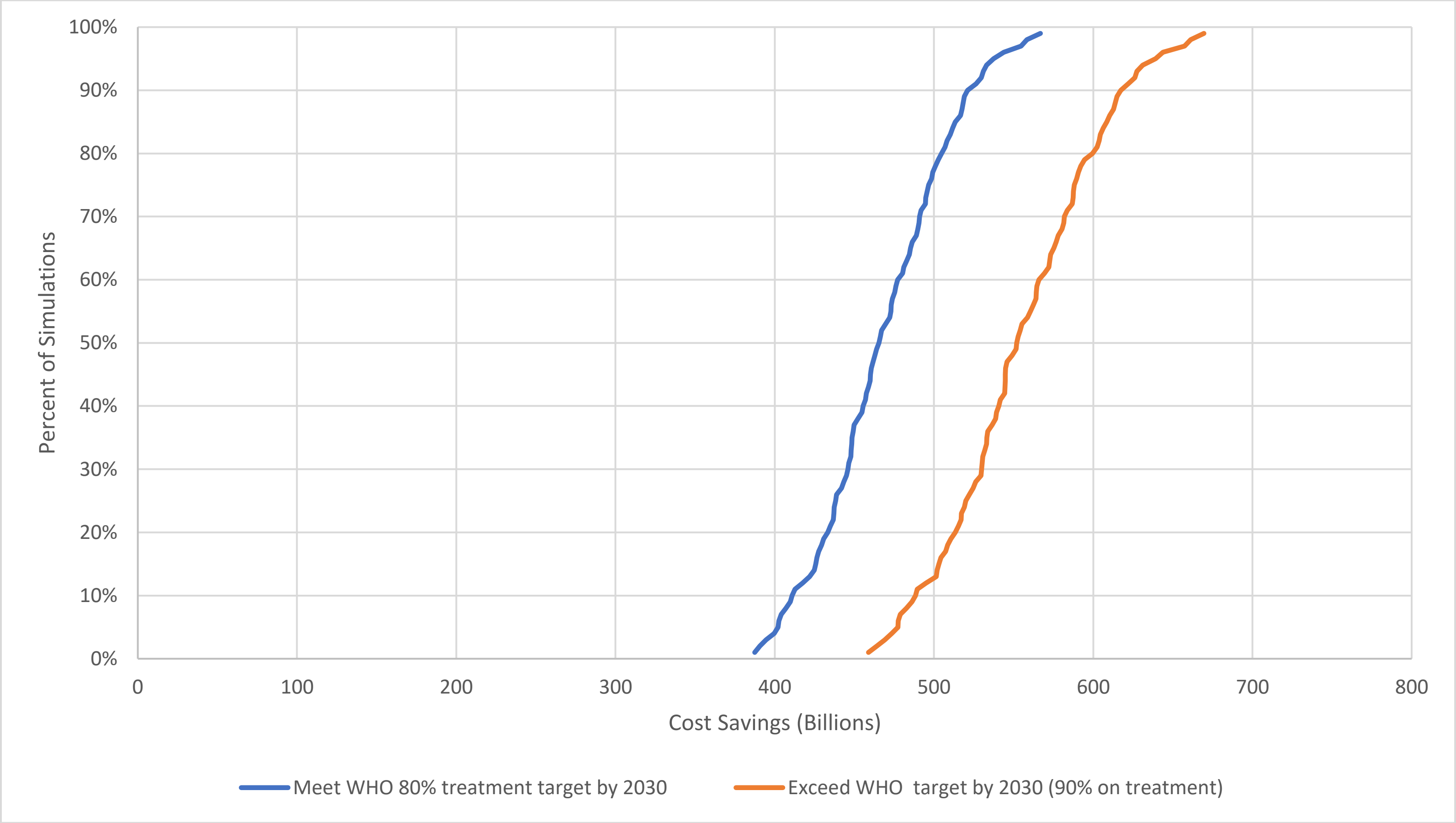

Appendix Figure S2b: QALYs Gained

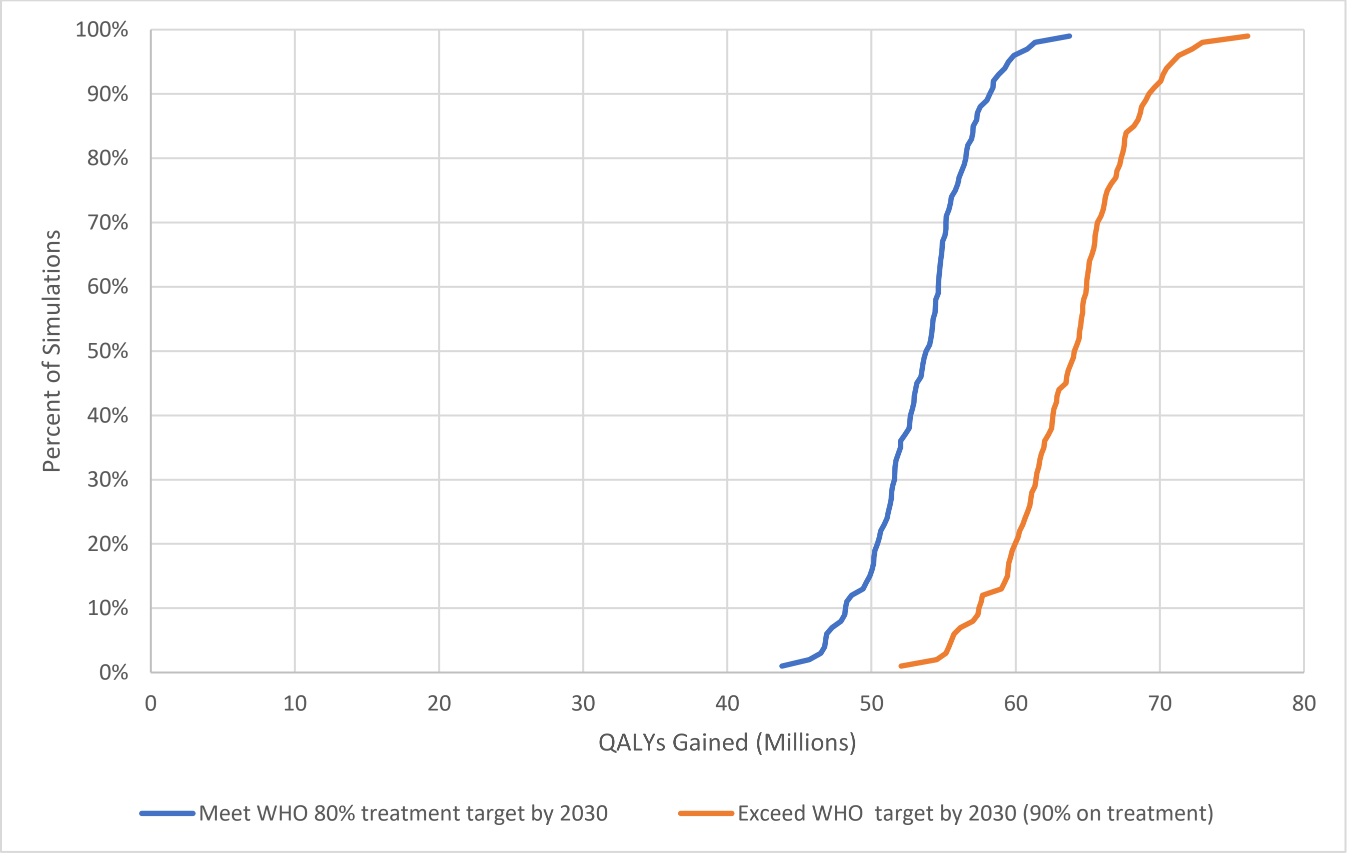

Supplement: Online Supplementary Document [file jogh-12-04043-s001.pdf]
